# Benchmarking microbial growth rate predictions from metagenomes

**DOI:** 10.1038/s41396-020-00773-1

**Published:** 2020-09-16

**Authors:** Andrew M. Long, Shengwei Hou, J. Cesar Ignacio-Espinoza, Jed A. Fuhrman

**Affiliations:** Department of Biological Sciences—Marine and Environmental Biology, University of Southern California, Los Angeles, CA USA

**Keywords:** Water microbiology, Microbial ecology

## Abstract

Growth rates are central to understanding microbial interactions and community dynamics. Metagenomic growth estimators have been developed, specifically codon usage bias (CUB) for maximum growth rates and “peak-to-trough ratio” (PTR) for in situ rates. Both were originally tested with pure cultures, but natural populations are more heterogeneous, especially in individual cell histories pertinent to PTR. To test these methods, we compared predictors with observed growth rates of freshly collected marine prokaryotes in unamended seawater. We prefiltered and diluted samples to remove grazers and greatly reduce virus infection, so net growth approximated gross growth. We sampled over 44 h for abundances and metagenomes, generating 101 metagenome-assembled genomes (MAGs), including Actinobacteria, Verrucomicrobia, SAR406, MGII archaea, etc. We tracked each MAG population by cell-abundance-normalized read recruitment, finding growth rates of 0 to 5.99 per day, the first reported rates for several groups, and used these rates as benchmarks. PTR, calculated by three methods, rarely correlated to growth (*r* ~−0.26–0.08), except for rapidly growing γ-Proteobacteria (*r* ~0.63–0.92), while CUB correlated moderately well to observed maximum growth rates (*r* = 0.57). This suggests that current PTR approaches poorly predict actual growth of most marine bacterial populations, but maximum growth rates can be approximated from genomic characteristics.

## Introduction

An organism’s growth rate is fundamental to its ecology and necessary to conceptually or mathematically model microbial community composition and dynamics. Therefore, many efforts have been made to estimate the growth rates of microbes from diverse ecosystems. Historically, these growth rate estimates have used time-course incubations of mixed communities, usually involving isotopic tracers of DNA or protein synthesis (reviewed in [1]). These approaches provide valuable information on the bulk growth of mixed communities. However, because they do not distinguish the contribution of individual phylogenetic groups from community-wide rates, they cannot be readily applied to native uncultivated microbes in a taxon by taxon manner in their natural habitats, which is often more germane to understanding ecological processes. Other growth rate estimations, such as ^18^O-labeled H_2_O stable isotope probing (e.g., [2]), do address the growth of individual taxa and require incubation experiments that may favor the growth of certain taxa over others due to bottle effects. To address individual, in situ growth rates, several culture-independent growth rate estimation methods have recently been developed that use intrinsic characteristics of microbial genomes and discrete metagenomic samples to estimate either a taxon’s maximum growth rate or take a snapshot of its growth rate, without incubation.

These recently developed genome-based growth estimates, codon usage bias (CUB) and peak-to-trough ratio (PTR), take two fundamentally different approaches that generate fundamentally different answers. CUB methods aim to predict maximum growth rates, while PTR approaches seek to approximate growth at the time of cell collection before DNA extraction via estimating DNA replication rates. CUB is based on the physiological strategy of cells, in which there is a tendency of highly expressed genes to prefer one set of codons corresponding to the most abundant tRNAs in the cell, while other genes are more likely to use alternative sets of codons (from the less abundant tRNAs) for the same amino acids. The degree of difference between the usage of abundant codons for highly expressed genes and alternative synonymous codons for the other genes is the amount of bias present in any genome. This CUB is more evident for cells that can grow faster and have a particularly high priority to make ribosomes to facilitate their fast growth. Viera-Silva and Rocha calculated the CUB of a wide range of prokaryotic taxa and found a strong correlation between observed maximum growth rates and CUB, which they leveraged to predict maximum growth rates by a multivariate approach [3]. Further, Kirchman found a strong correlation between observed and predicted maximum growth rates when applying this methodology to cultured marine taxa (including *Prochlorococcus*, SAR11, and others) in a recent review [1]. While CUB maximum growth rate estimators have been validated with pure cultures of organisms whose genomes are fully sequenced, there has been no validation on partial genomes from mixed natural communities. Further, maximum growth rates are often used in population modeling as a starting point, with growth reduced by limiting factors, which makes this validation all the more pertinent.

In contrast to CUB, PTR is an approach that is designed to work as an in situ measure of the actual growth rate of any prokaryote in any sample where genomic or metagenomic data are available. Thus, it can be remarkably powerful to interpret the growth status of microbes, and its attraction obvious due to the availability of ever increasing metagenomic and genomic datasets. This method is based on the observation that prokaryotes generally have circular genomes that are replicated bidirectionally from a fixed origin to a fixed terminus (opposite the origin). When the microbial genomes are fragmented and sequenced, as is done in metagenomics, the PTR of the population represented by a genome could be calculated from recruiting metagenomic reads across that genome. Populations more rapidly growing and replicating their DNA will be expected to have more reads recruited to and near the origin of replication (“peak”) compared to the terminus (“trough”). Following a simple conceptual model, the slope of the resulting read recruitment sine curve should be reflective of the growth of the population, with steeper slopes indicating faster growth rates; current PTR implementation methods aim at relative rather than absolute rates. The PTR method was first developed in practice by Korem et al. [4] for use with complete genomes and validated with *E. coli* growing in a chemostat. However, we do not have complete genomes for the large majority of prokaryotes in nature, and at best we often have partial genomes that are metagenomically assembled (MAGs) or single-cell amplified genomes. Such genomes are usually in many fragments and not only is the genomic order of those fragments unknown, but there is uncertainty about the locations of the origin and terminus. To address this problem, multiple methods modified the original PTR approach for use with incomplete genomes and MAGs. The first (iRep) was by Brown et al. [5], followed with a version for MAGs with low coverage (GRiD) by Emoila and Oh [6], and for MAGs with low coverage, low genomic completion, and high genomic redundancy (DEMIC) by Gao and Li [7]. While all three calculate PTR, one of their key differences is how they estimate the origin of replication and the terminus of an MAG. iRep uses coverage across overlapping genome fragments (windows) and then sorts the fragments from highest to lowest coverage to estimate the origin of replication and the terminus. Similarly, GRiD sorts genome fragments from highest to lowest but places fragments containing the *dnaA* gene at the origin and fragments with the *dif* gene near the terminus. Lastly, DEMIC infers the genome fragment placement using relative distances with a principal component analysis of fragment coverage in multiple samples.

All of the PTR approaches have shown strong relationships to growth rates when applied to pure cultures of various bacteria, using the data from either the Korem et al. study or from other bacterial growth studies [5–7]. However, these approaches have not been validated in complex microbial communities such as those found in marine surface waters. Such communities potentially include highly non-uniform populations of individuals with different recent histories and with many co-occurring close relatives. Furthermore, many such organisms have slow growth rates, with division times much longer than the minimum time it takes to replicate a genome, and with individuals probably growing at a variable rate over time (as unpredictable resources and/or inhibitors change). The strategies by which such cells manage DNA synthesis and other cell components in preparation for cell division under natural dynamic conditions are not known and could make applying PTR to such cells challenging.

Because a number of laboratories have been applying the PTR methods to field samples without these methods having been tested or validated in such conditions, we feel it is critical to attempt an evaluation of the approaches with natural mixed and non-clonal populations when growing in conditions that simulate important aspects of natural conditions, in our case, growth on the dissolved organic matter present in seawater. We also feel it is critical to evaluate CUB methods under these same conditions and with incomplete genomes, which would allow growth information from uncultivated microbes to be readily obtained in addition to their metabolic potential. Further, tests on pure cultures individually or in mixed mock communities, no matter how extensive or rigorous, would not fully address issues about applicability to mixed natural populations, especially when some of the problems in interpretation may be because of the heterogeneous nature of communities and natural microdiversity. Hence, we have manipulated natural mixed microbial communities in order to observe their growth while applying these metagenomic growth estimators.

The purpose of this study is twofold: (1) to test and potentially validate the CUB and PTR approaches using MAGs recovered from marine microbes growing in a complex community, and (2) to estimate potential growth rates of a broad variety of native planktonic marine microbes in natural dissolved organic matter. In these experiments, we grew bacteria in conditions meant to remove grazing and to eliminate as much viral infection as possible, so that the observed growth would reflect the actual growth rates in the bottles (not necessarily the ocean in situ rates, as conditions were manipulated). From these seawater dilutions, we used metagenomics to generate MAGs and assessed each MAG’s growth rate using the number of recruited reads normalized to direct cell counts, MAG completeness and cumulative contig length (equivalent to draft genome size), over the course of roughly 44 h. The estimated growth rates within these incubation experiments were then compared to CUB maximum growth rate predictions and “instantaneous” PTR-derived growth rate indices. Our analyses showed that CUB provided reasonable estimates of maximum growth rates, but PTR methods poorly predict actual growth rates of most taxa.

## Materials and methods

### Sampling and experimental design

Growth rate experiments were conducted in May and September of 2017. For each experiment, surface water was collected at 33°33′ N, 118°24′ W during the monthly sample collection of the San Pedro Ocean Time-series (SPOT). Bulk samples (>40 l) were collected on site and placed into coolers for transportation to USC. Upon arrival, bulk samples were first filtered through 80 μm nylon mesh (Sefar, Buffalo, NY, USA) and then sub-sampled into two pools. The first pool was pumped through Whatman® 47 mm 0.6 μm track-etch PC filters (GE Life Sciences, Marlborough, MA, USA) twice, retaining the filtrate, with a goal of a grazer-free sample (grazers are >0.6 μm and prokaryotes are mostly <0.6 μm). The second pool was pumped through a Prep/Scale-TFF Cartridge 30 kD 2.5 ft^2^ (Millipore, Billerica, MA, USA) and the virus-free filtrate/permeate was retained. These two pools were combined to dilute the 0.6 μm-filtered microbial communities to 9.8% in May and 9.5% in September. Duplicate 10 l samples were incubated in the dark (to minimize light-synchronized growth that can complicate PTR interpretation) in PC bottles at 17 °C in May (in situ 17.2 °C) and triplicate 10 l samples were incubated under the same conditions in September (in situ 20.4 °C). Total time from sampling until collection of the initial time point was ~6–8 h for both experiments. Samples were taken at 0, 12, 24, and 42 h in May and 0, 11, 20, 37, and 44 h in September for DNA extraction and cell counts. Cells were counted with the SYBR green method described by Noble and Fuhrman [8] in May and a modified Acridine Orange Hobbie et al. [9] method in September, also described by Noble and Fuhrman [8].

### DNA extraction, sequencing, assembly, and metagenome-assembled genome generation

In order to obtain enough DNA for sequencing, DNA was extracted from ~4 l for time 1, ~3 l for time 2, ~2 l for time 3, and ~1 l for time 4. A bulk sample (~1 l) was taken for DNA extraction from time 0 before dilution with virus-free seawater. Two (May incubation) and three (September incubation) biological replicates were extracted for each time point and sequenced separately. Water was pumped through 0.2 μm Durapore Sterivex^™^ filter units immediately after taken from the experimental vessels. DNA was extracted from Sterivex^™^ filter units using an All-prep® DNA/RNA minikit (Qiagen, Hilden, GR) with a modified protocol. Briefly, ~100 μl of combusted 0.1 mm glass beads (BioSpec Products, Bartlesville, OK, USA) were added directly to the Sterivex^™^ filter unit with lysis buffer from the All-prep® kit and mixed on a vortex mixer (VWR model VM-3000) for 20 min at the maximum setting, the liquid was retrieved from the filters and the manufacturer’s protocol was followed thereafter. The resulting DNA was processed for sequencing using Ovation® Ultra-low V2 DNA-Seq Library Preparation kits (NuGen, Tecan Genomics, Redwood City, CA, USA) with the manufacturer’s protocol using 100 ng of starting DNA and nine PCR cycles. DNA was sequenced on an Illumina HiSeq platform at the USC UPC Core Sequencing Facility (Los Angeles, CA, USA) using 2 × 250 bp chemistries.

The computer programs atropos v1.1.18 [10] and sickle v1.33 [11] were used to remove adapter sequences and bases with quality scores below 25, which was then verified with fastqc v0.11.5 [12]. All samples were assembled individually with metaSPAdes v3.12.0 [13] with a custom kmer set (-k 21,33,55,77,99,127) under the following subsampling regime: first, 1, 1.5, 2, 5, 10, and 20% of the reads were assembled separately, and then 5% of the remaining reads were assembled without replacement, followed by 10, 20, 33, and 50% of the reads, also without replacement (i.e., 5% of the unassembled reads were assembled and then 10% of the remaining reads were assembled without including reads that were assembled from the 5% step and so forth). These subassemblies were sorted into two sets based on contig length cutoff (2 kb) using seqkit v0.3.4.1 [14]. Those contigs shorter than 2 kb were assembled using Newbler v2.9 [15] with a minimum identity cutoff 0.98 (-mi 98) and a minimum overlap 80 nt (-ml 80), the resulting assembled contigs (≥2 kb) were then combined with the longer contig set. All these contigs longer than 2 kb were further co-assembled using minimus2 from the AMOS v3.1.0 toolkit [16] with a minimum identity 0.98 (-D MINID = 98) and a minimum length cutoff 200 nt (-D OVERLAP = 200). The co-assembled contigs were de-replicated using cd-hit v4.6.8 [17] with a 0.98 identity cutoff (-c 0.98), the de-replicated contigs were renamed and were used as references for read recruitment with bwa v0.7.15 [18] and the following metagenomic binning. MetaWRAP v1.1 [19] was used to bin contigs via MetaBAT v2.12.1 [20], CONCOCT v1.0.0 [21], and MaxBin2 [22] with a minimum length cutoff of 2 kb. The resulting bins were further refined within MetaWRAP without filtering for completion (-c 0) and allowing high contamination (-x 10,000). Anvi’o v5.1.0 [23] was also applied to bin contigs >5 kb using CONCOCT v1.0.0 proceeded by manual refinement with redundancy cut-offs of 2.5% for MAGs with 50–75% completeness, 5% for MAGs with 75–90 % completeness, and 10% for MAGs with >90 % completeness. In addition, Vamb v1.0.1 [24] and BinSanity v0.2.8 [25] were used to bin contigs with a minimum length cutoff of 4 kb. All bins generated by the six binners and the MetaWRAP refined bins were further refined using DAS_Tool v1.1.1 [26] with custom penalty parameters (--duplicate_penalty 0.4, --megabin_penalty 0.4) and a score threshold of 0.3. All the DAS_Tool refined bins were further refined manually using anvi’o v5.1.0 [23]. MAGs with at least 50% completion and fewer than 5% redundancy or at least 90% complete and fewer than 8% redundancy according to anvi’o were retained for further analysis. The GTDB taxonomic information of manually refined bins were predicted using GTDBTk v0.1.3 [27] and compared to NCBI taxonomy commonly used to provide more context to the previous literature where appropriate.

### Phylogenomic analysis

The bacterial and archaeal phylogenomic trees were constructed using GToTree v1.1.3 [28] and RAxML-NG v0.8.1 [29]. Briefly, GToTree uses Prodigal v2.6.3 [30] to predict the coding regions of the MAGs and uses HMMER v3.2.1 [31] to search for 74 bacterial and 76 archaeal universal single-copy marker genes. Then, these marker genes are concatenated and aligned using MUSCLE v3.8 [32]. Next, the alignments were trimmed using Trimal v1.4 [33] with the heuristic “-automated1” method. Both the bacterial and archaeal phylogenomic trees were constructed using RAxML-NG based on the GToTree produced trimmed alignments. RAxML-NG was run with ten randomized parsimony starting trees (--tree pars{10}), a fixed empirical substitution matrix, a discrete Gamma model with eight categories of rate heterogeneity, empirical amino acid state frequencies estimated from the sequence alignment (--model LG+G8+F), and was performed for 200 non-parametric bootstrap replicates (--bs-trees 200).

### Growth rate estimations

First, the relative abundance for each MAG was calculated from the number of reads that mapped to that specific MAG using bwa v0.7.15 [18] under the default parameters, which was then corrected with the completeness information from anvi’o v5.1.0 [23] and the length (in bp) of the MAG. This theoretical number of reads that map to each MAG was then divided by the total number of reads in each sample to calculate the relative abundance of that MAG. The completeness and genome length adjusted relative abundance of each MAG was multiplied by the cell count at each time point to estimate the absolute cell abundance for each MAG. This is essentially the same approach used by Brown et al. [4] to estimate the absolute abundance of individual species in infant fecal microbiomes from metagenomically derived relative abundances and cell counts.

Growth rates were calculated by both two-point and three-point (regression) calculations, as the slopes of ln-transformed MAG cell abundances over time. For most calculations, two-point rates were used in order to be consistent with the method of growth rate estimations used by Korem et al. [3], but we also did separate comparisons limited to when three-point regressions were significant. Observed maximum growth rates were taken from the highest estimates between the following time scales: 0–36 h and 18–42 h for May; 0–20 h, 11–37 h, and 20–44 h for September.

### Growth rate indices: codon usage bias and peak-to-trough ratio

The maximum growth rate of each MAG was predicted using a customized growthpred v1.0.8 (available at https://hub.docker.com/r/shengwei/growthpred) in metagenome mode (-m) and with universal codons (-c 0). Blast-retrieved ribosomal protein sequences were used as the highly expressed genes (-b) and compared to all the coding sequences of each MAG (-g).

PTR indices were calculated using iRep v1.10 [5], GRiD v1.3 [6], and DEMIC v1.0.2 [7]. iRep and GRiD were calculated for all MAGs >75% complete and DEMIC was calculated for every MAG. Briefly, the mapping information for each MAG was extracted from the previously generated bam files, then the iRep and GRiD indices were calculated based on the aligned paired reads, determined for: (1) the entire metagenomic dataset for that sample, and (2) reads from metagenomic fragments with specific ranges of insert sizes (100–350, 200–450, 300–550, and 400–650) to evaluate the effect of different insert sizes on the performance of PTR indices. In addition, GRiD was also run under the default parameters using each individual MAG as input. DEMIC was run under the default parameters using sam files generated with bowtie2 v2.3.0 [34].

### Statistical comparisons

To compare observed growth rates with CUB-predicted maximum growth rates, we used the highest overall growth rate from each taxon based on the time intervals mentioned above. For the PTR indices, the growth rates estimated from the time points before and after the point at which the PTR index was calculated were used for comparisons. For instance, if the PTR index was calculated from time point 1, the growth rate it was compared to was estimated using time points 0 and 2. For all comparisons, Pearson correlation coefficients and *p* values were calculated in R version 3.5.3 using the package Hmisc [35]. When a MAG had no apparent growth during the time frame, their corresponding CUB or PTR index values were removed before statistical analyses. Further, outliers as calculated using Tukey’s fences in PTR index values and their corresponding growth rates were excluded from statistical analyses.

## Results

### Metagenome-assembled genomes

**Fig. 1:**
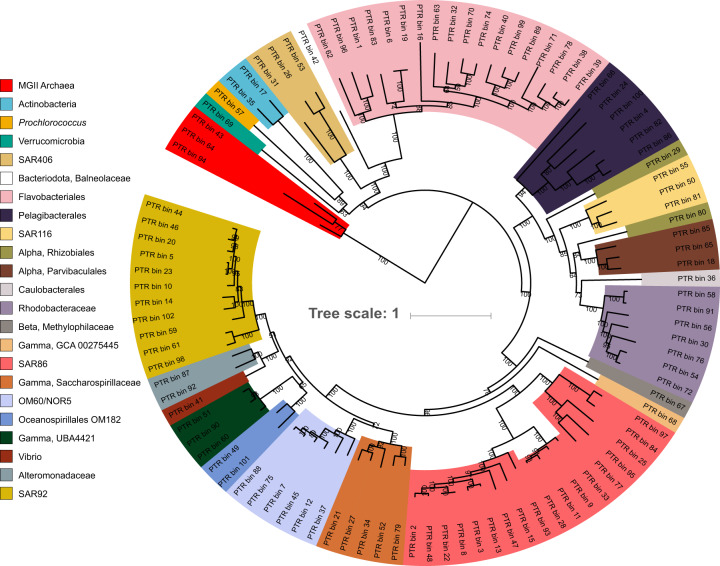
Phylogenomic tree of metagenomic-assembled genomes from our experiments. Numbers next to branches are bootstrap values (only bootstrap values ≥50 are shown here).

We recovered 101 MAGs that passed our quality criteria. The MAGs are on average 70% complete, 3% redundant, and cover most major groups of marine planktonic bacteria as well as MGII Euryarchaeota (Fig. 1). Several MAGs were from groups that have no cultivated representatives (Supplementary Table 1).

### Growth rate estimation from metagenome-assembled genomes

**Fig. 2:**
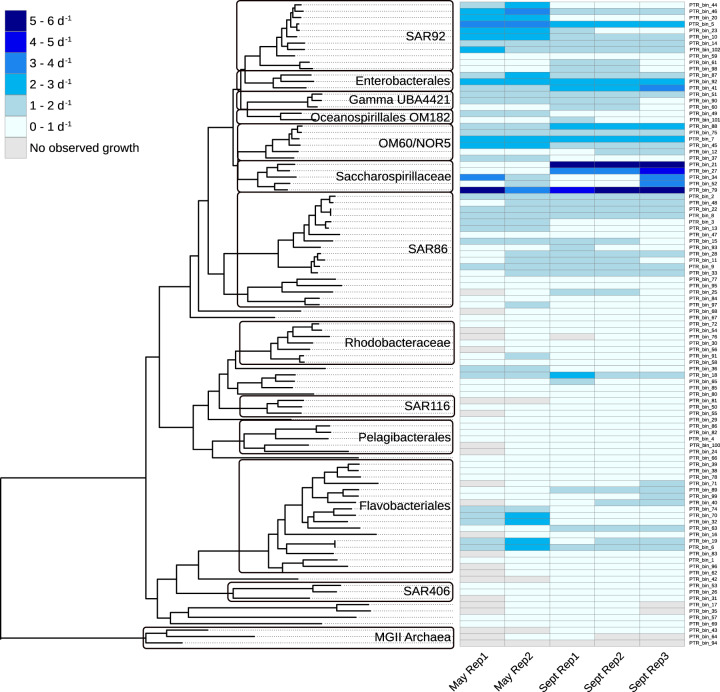
Phylogenomic tree with associated heatmap showing the highest observed growth for each taxon in each experiment and replicate. The detailed taxonomy information and observed growth rates can be found in Supplementary Table 1.

**Fig. 3:**
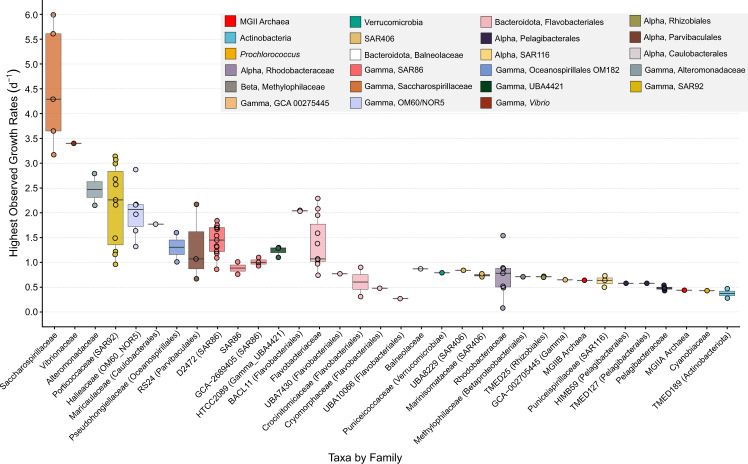
Box plot of the highest observed growth rate for each MAG, grouped by taxonomic family. The boxes are drawn from the 25th to the 75th quantiles and the center line of each box is the median. Whiskers indicate the smallest and largest values for each family.

Based upon read recruitment and cell counts, the overall range of highest estimated growth rates in the incubation bottles for all MAGs with detectable growth was 0.08–5.99 per day (Figs. 2 and 3). Oceanospirillales Saccharospirillaceae MAGs had the highest observed growth rates, of 3.17–5.99 per day. Among Pelagibacterales, the highest observed growth rates ranged from 0.40 to 0.58 per day. The majority (60 of 101) of MAGs had higher growth rates in the September experiment than in May (Fig. 2). However, many of the fastest-growing taxa grew faster in May, such as MAGs affiliated to SAR92 and Flavobacteriales.

### Highest observed growth rates compared to codon usage bias max growth rates

**Fig. 4:**
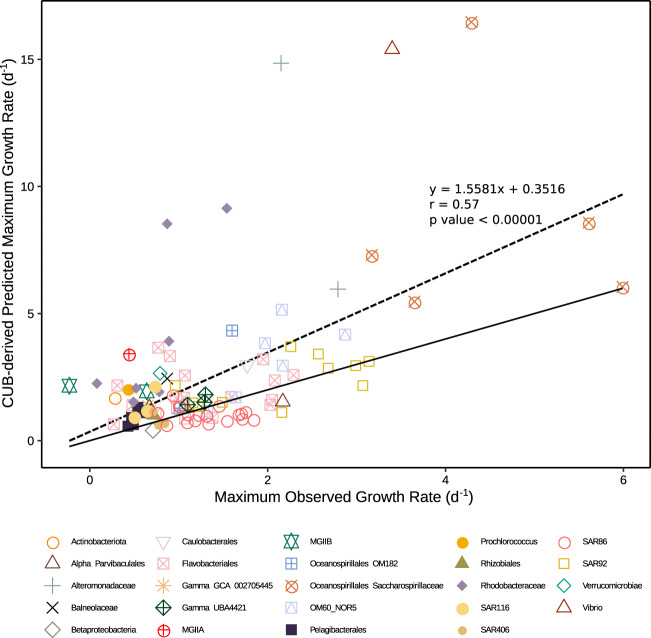
The highest observed growth rates, as measured over three time points, plotted against predicted maximum growth rates from codon usage bias predictor (growthpred). Solid line is *x* = *y*, so MAGs with symbols above the line grew more slowly than its predicted maximum, and below the line had faster growth than its predicted maximum. Note that when growth exceeded the predicted maximum, it was usually not by much. The dashed line is the linear regression between observed growth and predicted maximum growth, with the correlation, equation, and *p* value shown.

CUB-derived maximum growth rates had a range of 0.40–16.47 per day (Supplementary Table 1). The highest predicted maximum growth rates were from Oceanospirillales Saccharospirillaceae, Vibrionaceae, and Alteromonadaceae MAGs and the lowest were from Betaproteobacteria, Pelagibacterales, Actinobacteria, and SAR406 MAGs. Seventy-four of the 101 MAGs had a lower observed maximum growth rate during the experiments than a predicted maximum growth rate (Fig. 4). Pearson correlation analysis with predicted max growth rates and highest observed growth rates of all MAGs found a good correlation (*r* = 0.57, *p* < 0.00001, *n* = 101), especially considering the observed growth in the unamended filtered seawater we used is unlikely to be at or even near the organism’s maximum rate.

### Observed growth rates compared to peak-to-trough ratio indices

**Fig. 5:**
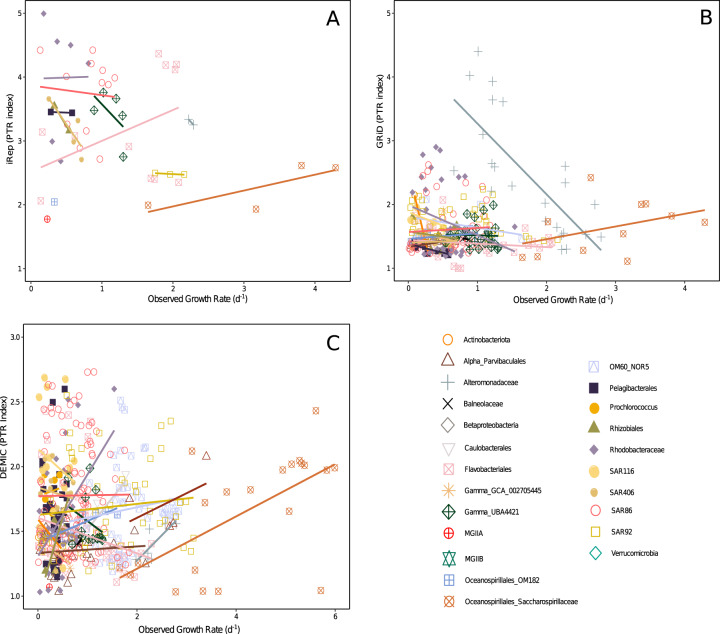
Observed growth rate, measured over three consecutive time points, plotted against peak-to-trough ratio indices at the middle time point. The PTR indices are **a** iRep, **b** GRiD, and **c** DEMIC. Lines are linear regressions for each taxon with more than two observations of both growth rates and PTR indices. Slopes should be positive and significant if the PTR index reflects growth. The similar extent of positive, flat, and negative slopes illustrates the poor general relationship between PTR and observed growth rates, with the exception of a few fast-growing Gammaproteobacteria (see text). Underlying data and regression statistics are in Supplementary Table 2.

The range of the three PTR indices were 1.69–4.99 (iRep, mean = 3.15), 1–4.4 (GRiD, mean = 1.63), and 1.01–12.82 (DEMIC, mean = 1.99). The correlations between PTR indices and observed growth rates of all taxa combined were either negative (iRep, *r* = −0.27, *p* value = 0.053, *n* = 52) or weak (GRiD, *r* = 0.077, *p* value = 0.20, *n* = 273; DEMIC, *r* = 0.072, *p* value = 0.13, *n* = 446). However, because PTR indices could have taxon-specific relationships with growth, we compared the observed growth rates and PTR values on a taxon-by-taxon basis. The overall picture showed many weak or even negative relationships, and very few taxa having a significant positive relationship between any PTR index and observed growth (Fig. 5 and all regression data shown in Supplementary Table 2). Only a few fast-growing Gammaproteobacteria were exceptions, particularly with the DEMIC PTR index. Oceanospirillales Saccharospirillaceae had significant correlations between observed growth rates and DEMIC (*r* = 0.63, *p* value = 0.0022, *n* = 21), and small sample sizes may have hampered statistical significance with iRep (*r* = 0.78, *p* value = 0.22, *n* = 4) and GRiD (*r* = 0.40, *p* value = 0.22, *n* = 11). Oceanospirillales OM182 growth also had significant correlations with the DEMIC index (*r* = 0.92, *p* value = 0.0004, *n* = 9), and a positive relationship with GRiD showing *r* = 0.49 (*p* value = 0.18, *n* = 9). The DEMIC index for these two taxa yielded the only significant positive correlations with observed growth when applying a Bonferroni-corrected alpha of 0.0025.

Measurable PTR indices were also found in MAGs that showed no detectable growth. Of 1166 calculable PTR indices, 304 occurred during times when the MAGs had no observable growth. Two different PTR indices calculated for the same MAG accounted for 128 of these 304 instances (Supplementary Table 3).

## Discussion

CUB and PTR growth estimation methods have previously been studied only with pure cultures, but for reasons we explained in the “Introduction”, there are questions about the applicability of such pure culture-based results to natural samples even though such organisms are typically the target of recent field research. Hence, we created a situation where such heterogeneous mixed communities would grow at rates similar to what we might expect in the field, on natural dissolved organics, and where we could track the growth of over a hundred MAGs, in order to apply these techniques and directly compare the results to measured growth rates. We reiterate that this is a validation approach for the methods, with highly manipulated samples, and not an attempt to measure the actual in situ rates of these taxa in the field at the time of the study.

### Codon usage bias maximum growth rate predictions

CUB-based maximum growth rate predictions had a significant statistical relationship with the observed MAG-derived maximum growth rate estimates (*r* = 0.57, *p* < 0.00001, *n* = 101). While Kirchman [1] applied this methodology to cultured organisms with complete genomes and found a similarly strong relationship, this is the first validation of the method using MAGs and their growth rates observed in a mixed microbial community. Note that MAGs are partial genomes at best and are very different from cultivated clonal genomes, in that they are necessarily somewhat chimeric representatives of natural populations that have some within-population diversity. The good relationship between the highest observed growth rates and predicted maximum growth rates suggests that CUB works reasonably well for prokaryotes that can be binned into a high-quality MAG (as defined in the “Methods”). These results allowed us to estimate at least the potential growth rates of members of ecologically important marine microbial subdivisions that have not yet been cultivated, such as SAR406, SAR86, and MGII Euryarchaeota. Note that as expected, organisms previously known to be adapted to oligotrophic environments, and with slow-growing cultured relatives, such as Pelagibacterales, had low predicted maximum growth rates (0.57–1.31 per day) and organisms with high growth rates in previous studies, such as *Vibrio* (up to 14 per day in [36]), had high predicted maximum growth rates (15.4 per day). These results confirm Kirchman’s [1] conclusion from cultured marine bacteria that CUB can be applied reasonably well for the estimation of maximum growth rates for many fast and slow-growing marine bacteria. We extend these results to incomplete genomes and at least one archaeal clade, and importantly to a variety of incomplete genomes and to uncultivated organisms. However, some slow-growing organisms, like *Prochlorococcus*, had a higher predicted maximum growth (2.05 per day) than any previous estimation of their growth rates from cultures (0.1–1 per day; e.g., [37–39]). Thus, while the relationship between observed growth rates and predicted growth rates across all taxa is strong, some taxa may have their growth potential overestimated with CUB. Others, as evidenced by the 27 of 101 MAGs with higher observed growth rates than predicted maximum growth rates, apparently have their growth potential underestimated, but few observed rates were very far from predictions. It is possible that CUB might be modified to more accurately predict maximum rates, by incorporating more training data from this and other similar experiments. CUB-based maximum growth rate predictors may be further improved by coupling metatranscriptomics with growth rate experiments to tailor highly expressed genes rather than using only ribosomal protein genes as is the case with growthpred, the predictor used in this study.

### Peak-to-trough ratio growth indices

In contrast to CUB, PTR indices as currently applied with MAGs did not work well for the vast majority of marine planktonic taxa we observed. Until PTR indices are tested and validated in other natural environments, we suggest results derived from these methods should be interpreted with caution. Several MAGs did not have any growth observed for parts of the experiment yet did have calculable PTR indices, suggesting not all PTR values are indicative of growing organisms. For taxa that did grow, the weak relationship between observed growth rates and all the variants of the PTR growth indices suggests that these methods poorly predict the growth of the large majority MAGs in the naturally derived microbial communities we observed. Oceanospirillales MAGs, the fastest-growing MAGs in the experiments, were a notable exception to this, suggesting PTR methods may be more suited to rapidly growing organisms. One reason may be that the very low initial Oceanospirillales MAG abundance and subsequent rapid growth suggest that Oceanospirillales MAGs were more genomically clonal and physiologically homogeneous than the organisms with high initial abundances and slow growth rates. Because one of the challenges in applying PTR to natural communities is cross-recruitment from close relatives when mapping reads, it is reasonable that a more clonal population would reduce the noise generated from such cross-recruitment. Co-occurrence of many close relatives is a characteristic frequently observed in natural populations, particularly the most abundant oligotrophs (e.g., [40, 41]). This relates to questions about the extent to which MAGs represent an amalgamation of closely related strains with potentially different growth rates. When MAGs represent highly microdiverse clusters of relatives that could potentially exhibit concomitantly diverse growth rates, it may lead to irregular and noisy read recruitment and less informative PTR index calculation.

Other challenges to the application of PTR include different microenvironments (rich versus less so) that individuals within a population could have been collected from, the extent some population members may be infected by viruses, the lack of knowledge on the physiology and replication strategy of many slow-growing microbes, and the extent of synchronization of the growth of particular populations. Synchronicity is common in phototrophs (diel cycles) and reported for at least some physiological processes even in oligotrophic heterotrophs like SAR11 [42], and it can also influence PTR interpretation.

We considered two methodological artifacts that may yield inaccurate PTR data. First, some MAG generation steps may merge related strains that might legitimately be considered part of a “population” but grow differently, yet all strains would recruit reads resulting in a noisy PTR calculation. To begin examining the effect of such merging, we altered our regular MAG generation protocol by eliminating the overlap assembly step that merges very close (but not identical) relatives, using only MAGs generated by one method (anvi’o). This should have merged fewer closely related strains into one MAG. Still, results from these MAGs yielded no better relationships between growth and PTR indices (Supplementary Fig. 1 and Supplementary Table 4). The second possible artifact relates to sequencing library preparation. Metagenomes today are most commonly generated with library preparation kits that involve a PCR-amplification step (i.e., linker amplified shotgun libraries), including this study and the dataset used to test all PTR indices [4]. It has been recently recognized that this amplification step alters the relative abundance of metagenomic reads compared to the original DNA, specifically yielding a small-insert bias due to amplifying and sequencing small inserts more readily than larger inserts [43]. This bias produces artifacts in quantitative read mapping, potentially altering PTR index values, or at least introducing considerable noise in the PTR estimation. We tried to avoid this artifact by sub-setting the read mapping files according to insert sizes (100–350, 200–450, 300–550, 400–650). We found that when we did so, PTR index values trended lower for the same MAG when using shorter insert sizes (Supplementary Table 5). However, the lowered PTR index values still produced weak and negative correlations when compared to observed growth rates and did not improve the PTR-based predictions of growth (Supplementary Table 2). We also considered how the method chosen for growth rate estimations might affect the relationship between growth and PTR index. The results we reported used all the two-point growth calculations, which allowed many separate determinations of growth and the maximum number of comparisons. If we restricted the comparisons to three-point regression-based growth estimates where the regression was significant, we had fewer comparisons, yet rates were about the same. This did not alter the relationship between growth and PTR indices, with correlations remaining weak and often negative (Supplementary Fig. 2 and Supplementary Table 2).

### Bacterial growth rates of uncultivated clades

While the primary aim of this study was to test PTR and CUB, the MAG-derived growth rate estimation approach itself allowed us to obtain growth information from clades without cultured representatives such as SAR406, SAR86, and MGII Euryarchaeota. This growth rate estimation method is based on the calculation of absolute abundances under the assumption of uniform DNA extraction efficiencies, as assumed previously in growth rate estimations using quantitative PCR [e.g., 44] and by Brown et al. [4] using a similar method to estimate absolute abundances of species within an infant fecal microbiome which were used to estimate the doubling time of *Klebsiella oxytoca*. It is encouraging that our calculation of growth rates for taxa with cultivated members fits in well with what is known from cultures; for example the ranges of both observed and predicted growth rates for the several *Pelagibacterales* MAGs were within the range of previously published observed growth rates from this group (0.4–0.6 per day; reviewed in [1]).

Even though the growth rate estimates are from manipulated conditions where grazers (along with in situ nutrient replenishment sources like phytoplankton) were filtered out and viruses are greatly reduced by dilution, they provide growth information on clades that would otherwise have none, and do reflect growth on natural dissolved organic matter in unamended seawater, thus meriting discussion. For example, the growth rates estimated for previously uncultivated SAR406 (0.29–0.39 per day) were similar to those of several other marine heterotrophs, such as MAGs belonging to Pelagibacterales and SAR116. The observed growth rates were lower but close in value to the CUB-predicted maximum growth rates. This result is interesting because SAR406 has usually been found at higher abundances in suboxic and hypoxic environments, leading researchers to think they preferred such conditions [45], and our conditions were fully aerobic. In addition, SAR86 MAGs also had a low range of both observed (0.53–1.84 per day) and predicted (0.64–1.82 per day) maximum growth rates. Most previous studies examining SAR86 growth also suggest low activity (e.g., [46–48]), except for a study in the coastal North Sea [49]. However, as SAR86 is thought to have at least three distinct subclades [50, 51], these previously published results may not be directly applicable to the SAR86 MAGs recovered in our study.

Two groups of MGII Euryarchaeota were present in our experiments: MGIIa and MGIIb. MGIIa are typically found in higher abundances in surface waters [52], whereas MGIIb are more prevalent in deeper waters (e.g., [53–55]). Despite the relatively low observed growth rates (0.19–0.44 per day), all three MGII MAGs had predicted maximum growth rates based on CUB near or over 2 per day. This suggests that MGII may be quite active in certain environments, as we previously reported for SPOT where MGII 16S rRNA sequences comprised over 40% of the microbial community (higher than SAR11) on one post-spring-bloom day [56]. Further, MGII have also been found to be very abundant in Monterey Bay, CA [57] and the Northern Gulf of Mexico “Dead Zone”, where they were found to be over 10% of the total microbial community [45].

## Conclusions

We studied bacteria and archaea, represented by MAGs, in mixed naturally derived marine communities grown under manipulated conditions that allowed us to track gross growth rates. We found they had growth rates that related fairly well to the CUB-based estimates of maximum growth rates, but not so well to metagenomic PTR-based estimates of in situ rates (except for the fastest growers). Assuming our results derived from marine plankton apply generally to other comparable habitats (at least), the data suggest that applying the CUB method to MAGs is likely to provide somewhat reasonable estimates of maximum growth rates, but that the current PTR methods do not reliably predict in situ growth rates. Looking forward, the CUB method could possibly be improved by an updated training dataset that considers more slowly growing organisms, many of which are now in culture. Other alterations to the CUB approach may include consideration of other genes that are known to be highly expressed in addition to ribosomal proteins. It is also possible that improvements in metagenomic assembly, binning, and read mapping, as well as better information on DNA replication strategies in diverse nutrient-limited slow-growing populations, may improve prospects for PTR-based growth estimates and other related approaches.

## Supplementary information

Supplemental Figure and Table Captions

Supplemental Figure 1

Supplemental Figure 2

Supplemental Table 1

Supplemental Table 2

Supplemental Table 3

Supplemental Table 4

Supplemental Table 5

## Data Availability

Raw reads and sample information have been submitted to NCBI under project ID PRJNA551656, the de-replicated assemblies and manually curated MAGs have been deposited at 10.6084/m9.figshare.9730628.
