# Supplementary material for: Benchmarking microbial growth rate predictions from metagenomes
Source: ISME J. 2020 Sep 16;15(1):183–95. doi: 10.1038/s41396-020-00773-1 (PMC7852909; doi:10.1038/s41396-020-00773-1)
Supplement: Supplementary file 1 — Supplemental Figure and Table Captions [file 41396_2020_773_MOESM1_ESM.docx]

***Supplemental Figures***

Supplemental Figure 1. Observed growth rate over three consecutive time points from MAGs generated without overlap-assemblers against peak-to-trough ratio indices. Lines are linear regressions for each taxon with more than two observations of both growth rates and PTR indices. As in Fig 5, slopes should be positive and significant if there is a relationship between PTR index and growth. The negative and non-significant slopes indicate that MAGs generated without an overlap assembler perform no better than MAGs that were generated with one (Fig 5). Underlying data and statistics are in Supplemental Table 5.

Supplemental Figure 2. Observed MAG growth rates from regressions fitted to three consecutive time points, compared to PTR indices (for regressions where p < 0.05). Underlying data and statistics are presented in Supplemental Table 2. The dashed line is the overall regression and the individual lines are regressions for individual taxa.

***Supplemental Tables***

Supplemental Table 1. MAG taxonomy, descriptive statistics (including length, completeness and redundancy), maximum observed growth rates, CUB-predicted maximum growth rates, PTR indices on the first tab. The second tab contains the normalized abundance of every MAG at every time point and replicate and the third tab contains sample metadata consisting of the metagenomic reads, total cell abundances, and time of each sample.

Supplemental Table 2. Statistical comparisons between growth rate estimations and PTR indices, each tab contains the information from one PTR index whether compared to growth rate estimates based on two-points or three-point regression or calculated with specific insert sizes (details of which are within the table). All growth rates from every MAG on additional tabs.

Supplemental Table 3. Calculable PTR index values during times with no observed MAG growth

Supplemental Table 4. PTR indices calculated with specific insert sizes, each tab contains the values for one PTR index with one range of insert sizes, details within.

Supplemental Table 5. MAG taxonomy and descriptive statistics for MAGs generated without overlap assembler, CUB predicted max growth rate and PTR indices on the first tab, percent of reads mapped to each MAG on the second tab, and statistical comparisons between growth rates and PTR indices on the remaining tabs, details within.
