# Supplementary figures and images for: Benchmarking microbial growth rate predictions from metagenomes
Source: ISME J. 2020 Sep 16;15(1):183–95. doi: 10.1038/s41396-020-00773-1 (PMC7852909; doi:10.1038/s41396-020-00773-1)

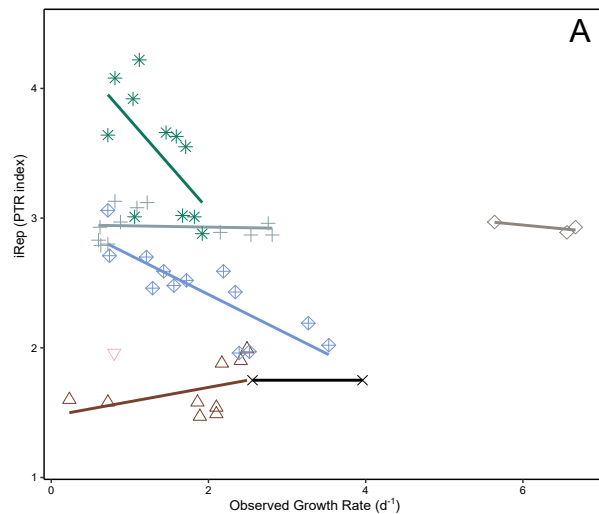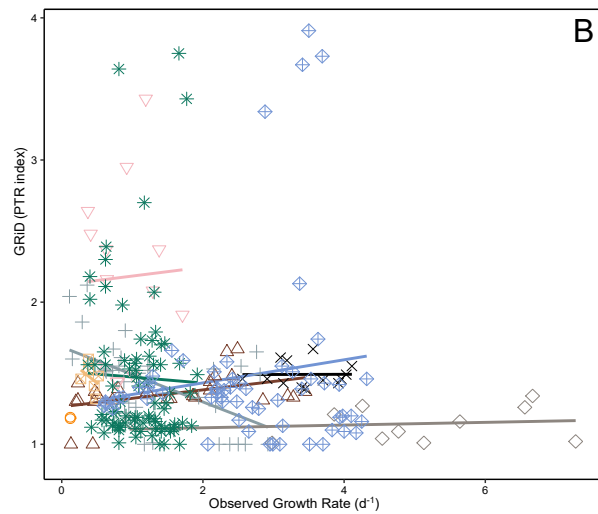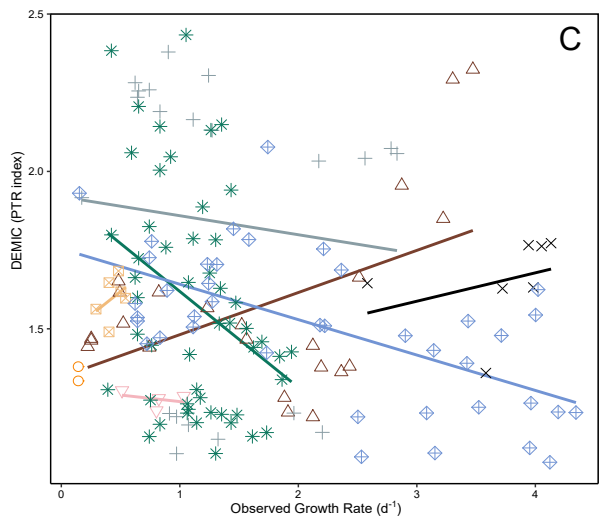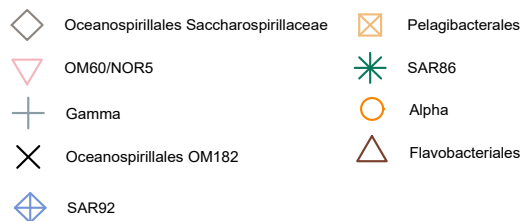

Supplement: Supplementary file 2 — Supplemental Figure 1 [file 41396_2020_773_MOESM2_ESM.pdf]
