# Supplementary material for: Benchmarking microbial growth rate predictions from metagenomes
Source: ISME J. 2020 Sep 16;15(1):183–95. doi: 10.1038/s41396-020-00773-1 (PMC7852909; doi:10.1038/s41396-020-00773-1)
Supplement: Supplementary file 3 — Supplemental Figure 2 [file 41396_2020_773_MOESM3_ESM.pdf]

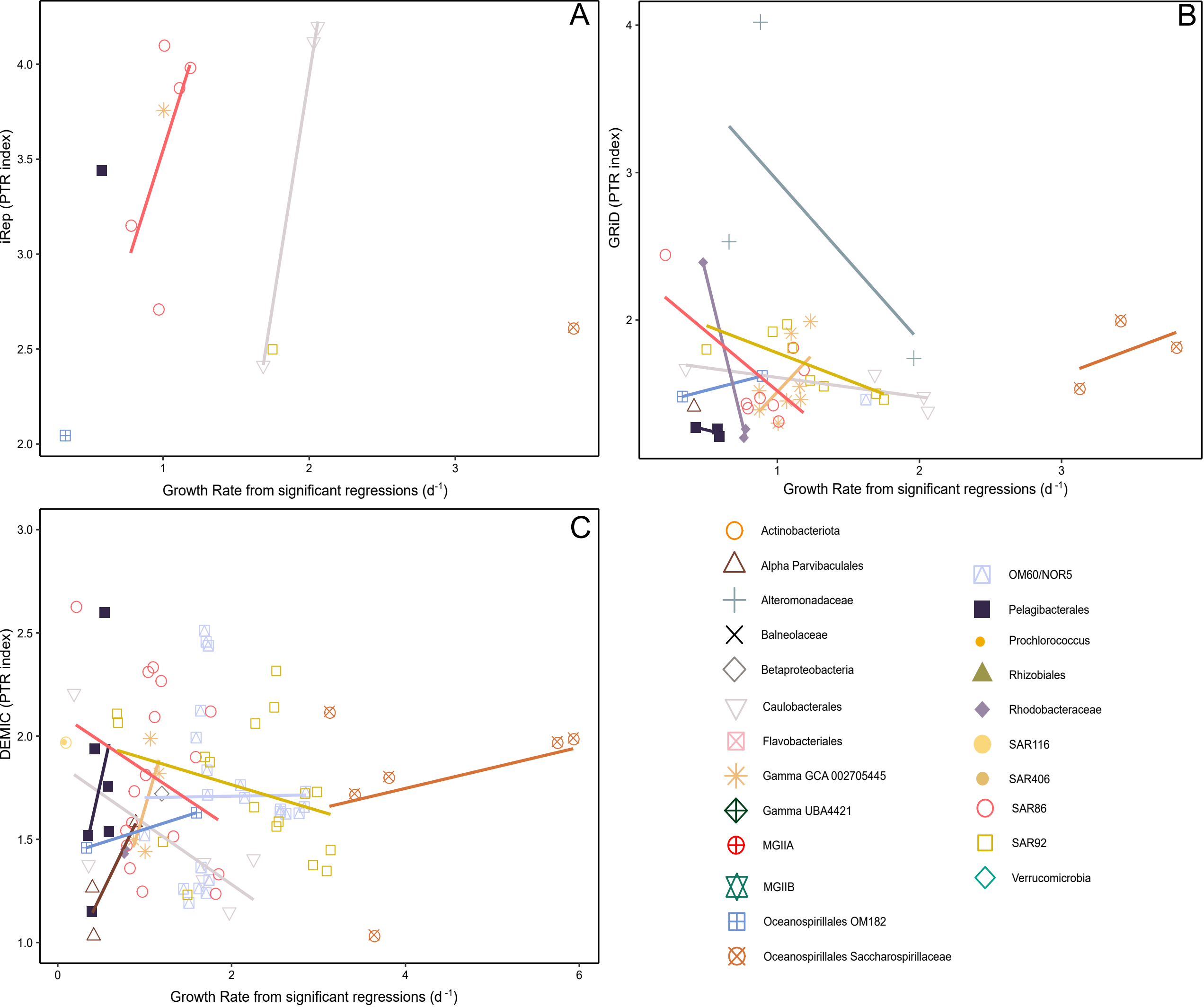

A
iRep (PTR index)
4.0
3.5
3.0
2.5
2.0
1
2
3
Growth Rate from significant regressions (d -1)
B
GRiD (PTR index)
4
3
2
1
2
3
Growth Rate from significant regressions (d -1)
C
DEMIC (PTR index)
3.0
2.5
2.0
1.5
1.0
0
2
4
6
Growth Rate from significant regressions (d -1)
Actinobacteriota
Alpha Parvibaculales
Alteromonadaceae
Balneolaceae
Betaproteobacteria
Caulobacterales
Flavobacteriales
Gamma GCA 002705445
Gamma UBA4421
MGIIA
MGIIB
Oceanospirillales OM182
Oceanospirillales Saccharospirillaceae
OM60/NOR5
Pelagibacterales
Prochlorococcus
Rhizobiales
Rhodobacteraceae
SAR116
SAR406
SAR86
SAR92
Verrucomicrobia
